# Increased Immune Complexes of Hypocretin Autoantibodies in Narcolepsy

**DOI:** 10.1371/journal.pone.0013320

**Published:** 2010-10-13

**Authors:** Aude Deloumeau, Sophie Bayard, Quentin Coquerel, Pierre Déchelotte, Christine Bole-Feysot, Bertrand Carlander, Valérie Cochen De Cock, Sergueï O. Fetissov, Yves Dauvilliers

**Affiliations:** 1 Digestive System and Nutrition Laboratory (ADEN EA 4311), Rouen Institute of Medical Research and Innovation, Federative Institute for Peptide Research (IFR23), Rouen University, Rouen, France; 2 Department of Neurology, Montpellier University Hospital Gui-de-Chauliac, National Reference Network for Narcolepsy, Montpellier, France; 3 Inserm U888, Montpellier, France; Brigham and Women's Hospital, Harvard Medical School, United States of America

## Abstract

**Background:**

Hypocretin peptides participate in the regulation of sleep-wake cycle while deficiency in hypocretin signaling and loss of hypocretin neurons are causative for narcolepsy-cataplexy. However, the mechanism responsible for alteration of the hypocretin system in narcolepsy-cataplexy and its relevance to other central hypersomnias remain unknown. Here we studied whether central hypersomnias can be associated with autoantibodies reacting with hypocretin-1 peptide present as immune complexes.

**Methodology:**

Serum levels of free and dissociated (total) autoantibodies reacting with hypocretin-1 peptide were measured by enzyme-linked immunosorbent assay and analyzed with regard to clinical parameters in 82 subjects with narcolepsy-cataplexy, narcolepsy without cataplexy or idiopathic hypersomnia and were compared to 25 healthy controls.

**Principal Findings:**

Serum levels of total but not free IgG autoantibodies against hypocretin-1 were increased in narcolepsy-cataplexy. Increased levels of complexed IgG autoantibodies against hypocretin-1 were found in all patients groups with a further increase in narcolepsy-cataplexy. Levels of total IgM hypocretin-1 autoantibodies were also elevated in all groups of patients. Increased levels of anti-idiotypic IgM autoantibodies reacting with hypocretin-1 IgG autoantibodies affinity purified from sera of subjects with narcolepsy-cataplexy were found in all three groups of patients. Disease duration correlated negatively with serum levels of hypocretin-1 IgG and IgM autoantibodies and with anti-idiotypic IgM autoantibodies.

**Conclusion:**

Central hypersomnias and particularly narcolepsy-cataplexy are characterized by higher serum levels of autoantibodies directed against hypocretin-1 which are present as immune complexes most likely with anti-idiotypic autoantibodies suggesting their relevance to the mechanism of sleep-wake cycle regulation.

## Introduction

Hypocretin-1 (orexin A) and hypocretin-2 (orexin B) are two neuropeptides produced by the same precursor molecule and synthesized in neurons of the lateral hypothalamus [1], [2]. Deficiency in hypocretin peptides production or defects in their receptors were found to cause narcolepsy-like symptoms in animals [3], [4]. In humans, narcolepsy with cataplexy (NC) is characterised by selective loss of hypocretin neurons in the brain with low levels of hypocretin in the cerebro-spinal fluid (CSF) [5], [6], [7]. Further evidence has accumulated supporting the causal role of hypocretin deficiency in the origin of NC [8], however, participation of hypocretin signaling in other forms of central hypersomnia including narcolepsy without cataplexy (NWC) or idiopathic hypersomnia (HI) is less understood, although a partial hypocretin deficiency is possible in the former condition [9], [10].

Selective loss or reduction of hypocretin neurons in NC together with the tight association with HLA DQB1*0602, the recent finding of polymorphisms in the T-cell receptor alpha locus and the presence of elevated Tribbles homolog 2 antibody levels suggest a possible autoimmune mechanism which so far remains elusive [11], [12], [13]. Several studies failed to provide evidence for a humoral autoimmune response against the hypocretin peptides [12], [14], [15]. However, transfer of total IgG autoantibodies (autoAbs) from patients with NC to mice supported the presence of functional autoAbs which might be relevant to NC [16], [17] and positive effect of intravenous IgG to normalize CSF hypocretin-1 level has been reported in an NC patient [18].

Failure to detect autoAbs response to the hypocretin peptides in NC might be related to the prevailing concept of autoAbs being the pure markers of autoimmune disease. However, another so far largely unexplored concept is to consider the presence of natural autoAbs reacting with self molecules including neuropeptides as a physiological phenomenon [19], [20]. Because any autoAbs exist as a free fraction and as immune complexes, it is possible that relative amount of free and complexed autoAbs against hypocretin peptides may participate in the regulation of hypocretin availability and therefore can be associated with sleep/wake dysregulation.

To address this question, in the present study, serum levels of free and dissociated (total) autoAbs reacting with hypocretin-1 peptide were measured in patients with central hypersomnias (including narcolepsy-cataplexy, narcolepsy without cataplexy and idiopathic hypersomnia) and compared to healthy subjects and to biological and clinical parameters relevant to sleep disorders.

## Materials and Methods

### Subjects

All subjects gave their written informed consent to participate in the study, which was approved by the Montpellier University Hospital's ethics committee. Eighty-two patients (41 men and 41 women, mean age 38.5±17.6) with chronic hypersomnias of central origin including thirty-nine subjects with narcolepsy with clear-cut cataplexy (NC), 17 with narcolepsy without cataplexy (NWC), and 26 with idiopathic hypersomnia (HI) with long sleep time participated in the study.

Diagnosis was made according to the revised International Classification of Sleep Disorders (ICSD-2). All patients were recorded for at least one night followed by the Multiple Sleep Latency Test (MSLT) the next day consisting of five naps scheduled at 2-h intervals starting at 9:00 h [21]. None of the patients were taking psychostimulants for at least two weeks or anticataplectic medications or any other medication known to influence sleep or motor activity for at least one month prior to the sleep laboratory recording. Patients were systematically evaluated for clinical parameters including: disease duration, Epworth Sleepiness Scale (ESS), cataplexy frequency scale (from 0 to 5) [22], hypnagogic hallucinations, sleep paralysis, and body mass index (BMI).

Narcolepsy with cataplexy was diagnosed based on the presence of excessive daytime sleepiness (EDS) and cataplexy, HLA DQB1*0602 positivity and of at least two sleep onset REM periods (SOREMPs) during the MSLT. The frequency of cataplectic attacks was evaluated on a scale from 1 to 5 as previously published [22]. Narcolepsy without cataplexy was diagnosed based on the presence of EDS, mean sleep latency below 8 min and at least two SOREMPs during the MSLT. Idiopathic hypersomnia with long sleep time was characterized by a complaint of constant EDS (ESS >12) and non refreshing naps irrespective of their duration (>1 hour duration), an uninterrupted and prolonged night-time sleep (>10 hours), sleep inertia and sleep efficiency on the polysomnography above 90%. It should be noted that 15 patients out of 26 affected with HI presented mean sleep latency below 8 minutes on the MSLT. A diagnosis of psychiatric disease according to DSM-IV [23] criteria was ruled out as it was for obstructive sleep apnea syndrome on the basis of the polysomnographic recording. All patients with an index of respiratory events (apneas + hypopneas) greater than 10 or with periodic leg movements index during sleep greater than 10 (except for narcolepsy with cataplexy) were excluded from the study.

Twenty-five non overweight subjects (10 men and 15 women, mean age 45.2±16.7) were recruited as normal controls. All controls were community-dwelling adults recruited from local associative networks. Exclusion criteria were a positive history of neurological or psychiatric disease, and/or the presence of sleep complaint including EDS (none of them had an ESS score above 10).

### Blood and CSF sampling

Both patients and controls underwent fasting venous blood sampling between 7 to 8 AM with similar procedures at the Montpellier Sleep Disorder Center. Sera were separated and frozen immediately for further analysis and were handled similarly between patients and controls before assay. A lumbar puncture was performed in 35 patients (19 NC, 7 NWC, 9 HI) in order to measure the CSF hypocretin-1/orexin A levels. CSF samples were collected between 5 to 7 PM and stored immediately at −80°C until use. Hypocretin-1 peptide levels were determined in duplicate from CSF samples without prior extraction using ^125^I radioimmunoassay kits from Phoenix Pharmaceuticals, Inc. (Belmont, CA), according to the manufacturer's instructions. The detection limit was 10 pg/ml and intra-assay variability was less than 10%. CSF hypocretin-1 levels of less than 110 pg/ml were classified as low, between 110 and 200 pg/ml as intermediate and above 200 pg/ml as normal [24].

### Hypocretin peptide autoantibody assay

Serum levels of IgG, IgM and IgA autoAbs reacting with hypocretin-1 peptide in all patients and controls were measured using enzyme-linked immunosorbent assay (ELISA) technique. The measurements were performed blinded for the diagnostic phenotype. The hypocretin-1 peptide (Bachem AG, Bubendorf, Switzerland) was coated on Maxisorp plates (Nunc, Rochester, NY) using 100 µl and a concentration of 2 µg/ml in 100 mM NaHCO_3_ buffer, pH 9.6 for 72 h at 4°C. Plates were washed (3 times) in phosphate-buffered saline (PBS) with 0.05% Tween 20, pH 7.4, and then incubated overnight at 4°C with 100 µl of human sera diluted 1∶200 in PBS to determine free autoAbs levels or diluted 1∶200 in dissociative 3M NaCl, 1.5 M glycine buffer, pH 8.9 to determine total autoAbs levels. The optimal dilutions of sera (1∶200) were determined by several dilutions (1∶100, 1∶200, 1∶400) to obtain for the same dilution mean OD levels of both free and total autoAbs in the linear range of ELISA. The plates were washed (3×) and for the detection of IgG, IgM or IgA classes of autoAbs incubated with 100 µl (1∶2000) of rabbit anti human IgG, anti human IgM or anti-human IgA antibodies, respectively, all conjugated with alkaline phosphatase (Sigma, St. Louis, MO) for 3 h at room temperature. Then, following washing (3×), 100 µl of p-nitrophenyl phosphate solution (Sigma) was added as alkaline phosphatase substrate. After 40 min of incubation at room temperature, the reaction was stopped by adding 3N NaOH. The optical density (OD) was determined at 405 nm using a microplate reader. Blank OD values resulting from the reading of plates without addition of human sera were subtracted from the sample OD values. Each determination was done in duplicate. The variation between duplicate values was less than 5%. Presence of hypocretin-1 autoAbs in immune complexes was estimated by ratios between levels of total (dissociated) and free autoAbs and their percentage relative to the total levels of autoAbs was calculated.

### Hypocretin autoantibody purification

Total IgG was purified from 0.5 ml of serum samples from eleven randomly selected NC patients using Immobilized Protein G Plus (Pierce, Rockford, IL) according to manufacturer instructions. Presence of IgG in the eluates was confirmed by western blot with peroxidase-conjugated goat anti-human IgG antibodies (Dako, Copenhagen, Denmark).

Individual samples of total IgG purified from patients' sera were combined into one pool which was further purified using affinity chromatography with hypocretin-1 peptide (Bachem) coupled to the pre-activated beads according to manufacturer's instructions (UltraLink, Pierce). Purified IgG were concentrated by lyophilization and dilution to 50 µg/ml in water. Presence of IgG autoAbs reacting with hypocretin peptides was verified by western blot (see below) on a rat hypothalamic homogenate.

### Western blot

To validate by western blot the presence of hypocretin reactive IgG autoAbs in affinity purified samples from NC patients, two Wistar rats were killed by decapitation and entire blocks of the hypothalamus were dissected and placed into ice-cold lysis buffer containing 0.1% protease inhibitor cocktail (Sigma). Tissues were manually homogenized. Vials were placed on ice for 15 min and then centrifuged for 15 min at 4°C and 12,000 rpm. The supernatant containing proteins was collected and stored at −80°C until analysis. Protein samples in duplicates (25 µg, 50 µg and 75 µg) were separated on 20% acrylamide SDS gel in Tris-Glycine buffer and transferred to a nitrocellulose membrane (GE Healthcare, Orsay, France), which was blocked for 1 h at room temperature with 5% (w/v) non-fat dry milk in TBS (10 mmol/L Tris, pH 8; 150 mmol/L NaCl) plus 0.05% (w/v) Tween 20. Then, the membrane was cut into two parts and each part was incubated overnight at 4°C with human hypocretin-1 affinity purified IgG autoAbs (1∶10) or with rabbit anti-hypocretin-1 IgG antibody (1∶1000, Bachem). After three washes in a blocking solution of 5% (w/v) non-fat dry milk in TBS/0.05% Tween 20, membranes were incubated for 1 h with goat anti-human IgG (1∶5000, Dako) or anti-rabbit IgG (1∶5000, SantaCruz Biotechnology) secondary antibodies, respectively, both peroxidase-conjugated. After three washes, the peroxidase reaction was revealed using the ECL detection kit (GE Healthcare). Protein bands were compared with the molecular weight standard (Precision Plus, BioRad) and films were scanned using ImageScanner III (GE Healthcare). For negative controls, after protein transfer in a separate set of membranes, only secondary antibodies were applied and revealed.

### Anti-idiotypic autoantibody assay

Serum levels of anti-idiotypic IgM autoAbs reacting with anti-hypocretin-1 IgG autoAbs affinity-purified from NC patients' sera were measured in all patients and controls using ELISA technique as described above with some modifications. Briefly, patients' anti-hypocretin-1 IgG autoAbs were coated on Maxisorp plates (Nunc) at 2 µg/ml in 100 mM NaHCO_3_ buffer; sera (diluted 1∶100 in PBS) from patients and controls were applied overnight and presence of bound IgM autoAbs was detected using rabbit anti-human IgM antibodies conjugated to alkaline phosphatase (1∶2000, Sigma) which was developed as described above.

### Statistical analysis

Data were analyzed and graphs were plotted using the GraphPad Prism 5.02 program (GraphPad Software Inc., San Diego, CA). Levels of autoAbs were compared using ANOVA or Kruskal-Wallis tests according to normality tests and post hoc Tukey-Kramer or Dunn's tests were correspondingly performed. NC group was additionally compared to other groups using the Student's t-test or the Mann-Whitney (MW) test according to normality test. Correlations between autoAbs levels and clinical parameters were calculated using the Spearman two-tail test. Bonferroni correction for multiple tests was applied by multiplying p-values by 9 (number of clinical parameters tested). Clinical parameters were compared between the patient's groups using ANOVA, Kruskal-Wallis or Chi-square tests. In all cases, p<0.05 was considered to be statistically significant.

## Results

### Clinical and biological characteristics

Clinical and biological characteristics of patients with central hypersomnia are presented in Table 1. Patients' groups were not significantly different with regard to age, BMI, and ESS scale. There were gender differences between hypersomnia populations, with more males in the NC group compared to both HI and NWC groups. As expected, the presence of hypnagogic hallucinations, sleep paralysis, number of SOREMPs, mean sleep latency and CSF hypocretin-1 levels differ between the patient groups. Low CSF hypocretin-1 levels, i.e. below 110 pg/ml was found in 38 among 39 patients with NC (one patient was within the intermediate range i.e. 132 pg/ml) and none in NWC (one patient was within the intermediate range, i.e. 148 pg/ml) or in HI patients.

**Table 1:** Clinical and biological characteristics of patients with central hypersomnia including narcolepsy-cataplexy (NC), narcolepsy without cataplexy (NWC) and idiopathic hypersomnia (HI).

*Patients characteristics*	NC N = 39	NWC N = 17	HI N = 26	p-values
Males/Females	28/11	5/12	8/18	p<0.001a,b
Age, years	40.4±18.9	37.9±18.9	36.4±14.8	p = 0.65
Disease duration, years	21.2±18.8	19.1±17.6	15.4±13.6	p = 0.47
BMI, (kg/m^2^)	25.8±4.3	23.9±3.6	24.1±4.3	p = 0.14
ESS score	18.6±3.5	16.2±4.1	17.3±3.8	p = 0.08
Cataplexy score	3.0±1.5	0	0	
Hallucinations, %	65.8	35.3	25.9	p = 0.004a,b
Sleep paralysis, %	47.4	23.5	11.1	p = 0.007a,b,c
**MSLT**				
SOREMP, n	3.5±1.2	3.2±1.7	0.3±0.5	p<0.0001b,c
Mean sleep latency, min	5.2±2.9	5.6±2.8	7.7±3.9	p = 0.01b,c
**CSF hypocretin-1**, Mean levels (pg/ml) Low levels (<110 pg/ml), %	N = 19 33.4±71.8 94.7	N = 7 349±114.80	N = 9 458±140 0	p<0.0001^b^

Significant differences,

**^a^**NC vs. NWC,

**^b^**NC vs. HI,

**^c^**NWC vs. HI.

### Hypocretin-1 autoAbs assay

**Figure 1:**
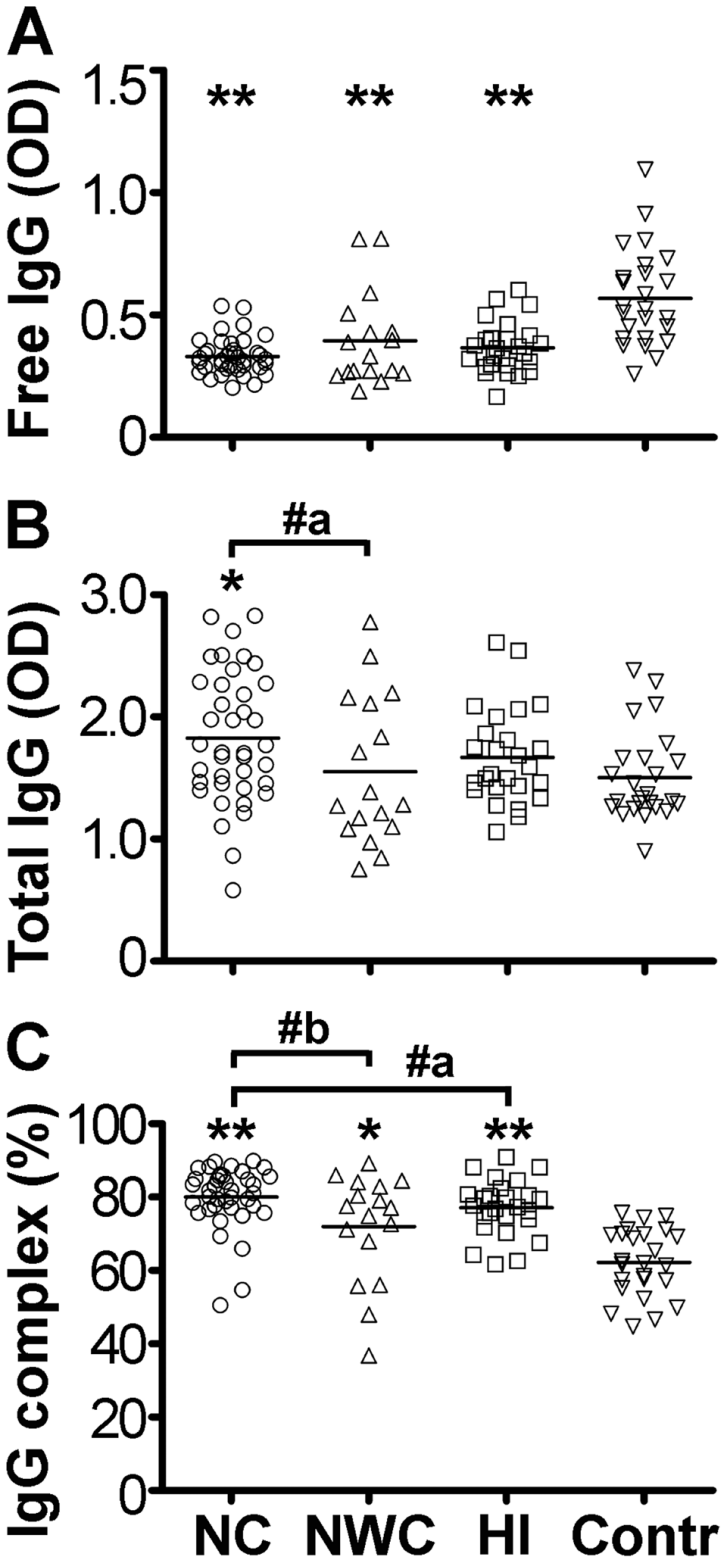
Serum levels of free (A), total (B) and percentage of immune complexes (C) of IgG autoAbs reactive with hypocretin-1 in subjects with central hypersomnia and controls. Bars show medians, *p<0.05, **p<0.01 Dunn's tests vs. controls. #a, p<0.05, two-tails, #b, p<0.05, one-tail, Mann-Whitney -tests.

Serum levels of free IgG autoAbs directed against hypocretin-1 were lower in all three groups of patients with central hypersomnias than in controls (Kruskal-Wallis test p<0.0001, Fig. 1A). In contrast, levels of hypocretin-1 total IgG autoAbs were not lower, and even higher in the NC group as compared to controls and NWC (Fig. 1B). These changes in the levels of total and free IgG autoAbs resulted in a higher percentage of these autoAbs present in immune complexes in all patients groups (median ±SD, NC, 82±8%, NWC, 77±15%, HI, 77±8% and controls 62±9%, Kruskal-Wallis test p<0.0001, Fig. 1C). Moreover, the levels of immune complexes of hypocretin-1 autoAbs were significantly higher in the NC group comparative to other central hypersomnias (Fig. 1C).

**Figure 2:**
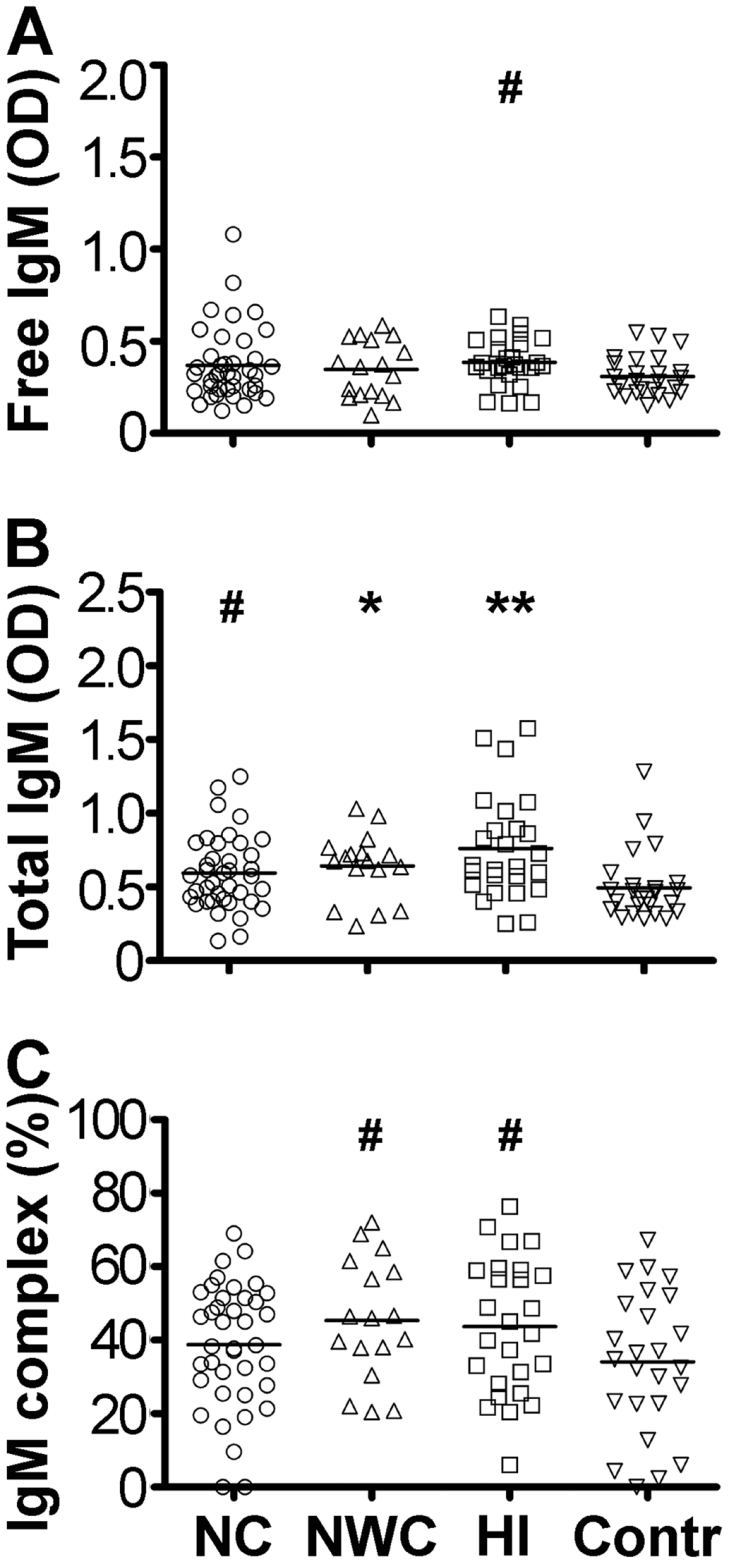
Serum levels of free (A), total (B) and percentage of immune complexes (C) of IgM autoAbs reactive with hypocretin-1 in subjects with central hypersomnia and controls. Bars show medians, *p<0.05, **p<0.01 Dunn's tests vs. controls. # p<0.05 two-tails, Mann-Whitney -test vs. controls.

Serum levels of free IgM class of autoAbs directed against hypocretin-1 were found elevated in the HI group vs. controls (Fig. 2A). Levels of total IgM autoAbs directed against both peptides were increased in all patients with central hypersomnia and this increase was more pronounced in the HI group (Fig. 2B). Increased percentage of hypocretin-1 IgM autoAbs present in immune complexes was found in the NWC and HI groups comparative to controls (Fig. 2C).

**Figure 3:**
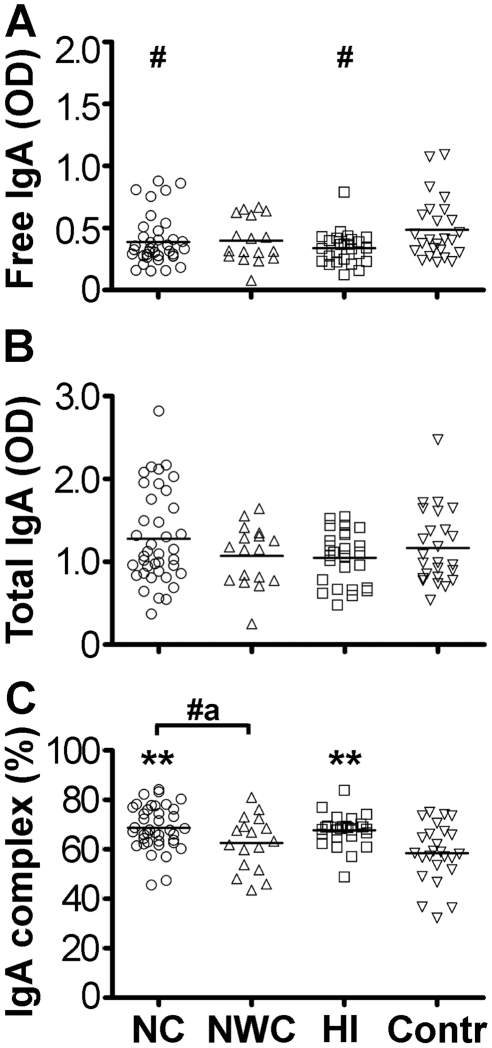
Serum levels of free (A), total (B) and percentage of immune complexes (C) of IgA autoAbs reactive with hypocretin-1 in subjects with central hypersomnia and controls. Bars show medians, **p<0.01 Dunn's tests vs. controls. # p<0.05, two-tails, Mann-Whitney-tests vs. controls and #a, p<0.05, one-tail Mann-Whitney test.

Serum levels of free IgA class of autoAbs directed against hypocretin-1 were lower in the NC and HI groups vs. controls (Fig. 3A). No significant differences in levels of total IgA autoAbs were detected (Fig. 3B). More immune complexes of hypocretin-1 IgA autoAbs were found in the NC and HI groups vs. controls (Fig. 3C).

### Correlations of hypocretin-1 autoAbs with clinical parameters

#### Disease duration

After correction for multiple tests, we found significant negative correlations between disease duration and levels of hypocretin-1 free IgG autoAbs (*r = *−0.35, p = 0.013), and levels of hypocretin-1 free IgM autoAbs (*r = *−0.39, p = 0.003). In contrast, disease duration correlated positively with the IgA class of total and free hypocretin-1 autoAbs (*r* = 0.4, p = 0.002, and *r* = 0.31, p = 0.04, respectively).

#### Body mass index (BMI)

After correction for multiple tests, no significant correlations were found between BMI and hypocretin-1 autoAbs levels. However, before correction, BMI correlated negatively with levels of hypocretin-1 free IgG in patients with central hypersomnia combined (*r = *−0.28, p = 0.01) and in the NC group (*r = *−0.36, p = 0.02).

#### Cataplexy

No significant correlations were found in NC subjects between the frequency of cataplexy and hypocretin-1 autoAbs. The presence of IgG immune complexes with hypocretin-1 correlated positively with the frequency of cataplexy (*r* = 0.24, p = 0.03) before Bonferroni correction.

#### Daytime sleepiness

No significant correlations were found between the ESS score or mean sleep latency and hypocretin autoAbs after correction for multiple tests. Significant correlations for ESS with levels of hypocretin-1 free IgG autoAbs in the NC group (*r* = 0.39, p = 0.016), or mean sleep latency with levels of immune complexes of hypocretin-1 IgA autoAbs (*r* = 0.35, p = 0.035) were found before corrections in patients with NC.

#### Hallucinations, sleep paralysis and sleep-onset REM periods (SOREMP)

No significant correlations between hypnagogic hallucinations, sleep paralysis and SOREMP with hypocretin-1 autoAbs were found in the NC and NWC groups or all groups of patients combined.

#### CSF hypocretin-1 levels

No significant correlations were found between CSF hypocretin-1 levels and hypocretin-1 autoAbs after correction for multiple tests. However, before correction, CSF hypocretin-1 levels correlated negatively with free IgG autoAbs against hypocretin-1 in the NC group (*r* = −0.36, p = 0.04).

#### Western blot

**Figure 4:**
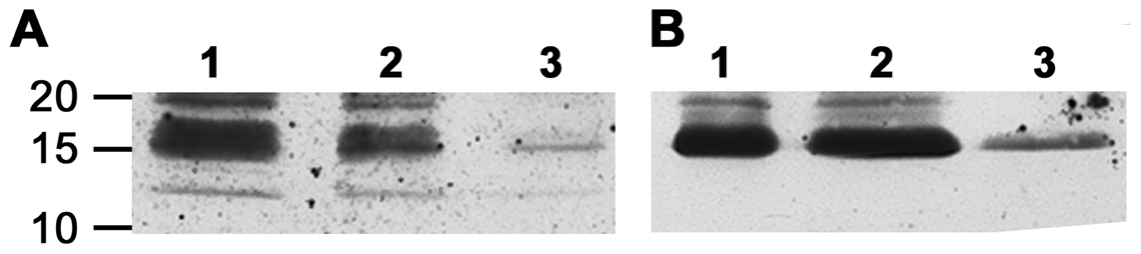
Western blot detection of a band corresponding to the hypocretin precursor protein (16 KDa) in the rat hypothalamic homogenate. **A**. Detection using human IgG autoAbs affinity purified for hypocretin-1 peptide from sera of NC patients. **B**. Detection using commercial rabbit anti-hypocretin-1 antiserum. Columns 1, 2 and 3 correspond to 75, 50 and 25 µg, respectively, of protein amount from the hypothalamic homogenate loaded into the gel. Molecular weight markers (KDa) are shown on the left.

Using hypocretin-1 reactive IgG autoAbs affinity purified from sera samples of NC patients we were able to detect a band of approximately 16 KDa on western blot of the rat hypothalamic homogenate (Fig. 4A). The band of the same size was detected using a commercial rabbit anti-hypocretin-1 antiserum (Fig. 4B) which was previously shown to correspond to the hypocretin precursor protein [25]. The intensity of the band corresponded to the amount of protein homogenates. In a negative control experiment, when no NC patient's affinity purified hypocretin-1 IgG autoAbs or rabbit anti-hypocretin-1 IgG antibody were added, no bands corresponding to molecular weight of 16 KDa were detected.

#### Anti-idiotypic IgM autoantibodies

**Figure 5:**
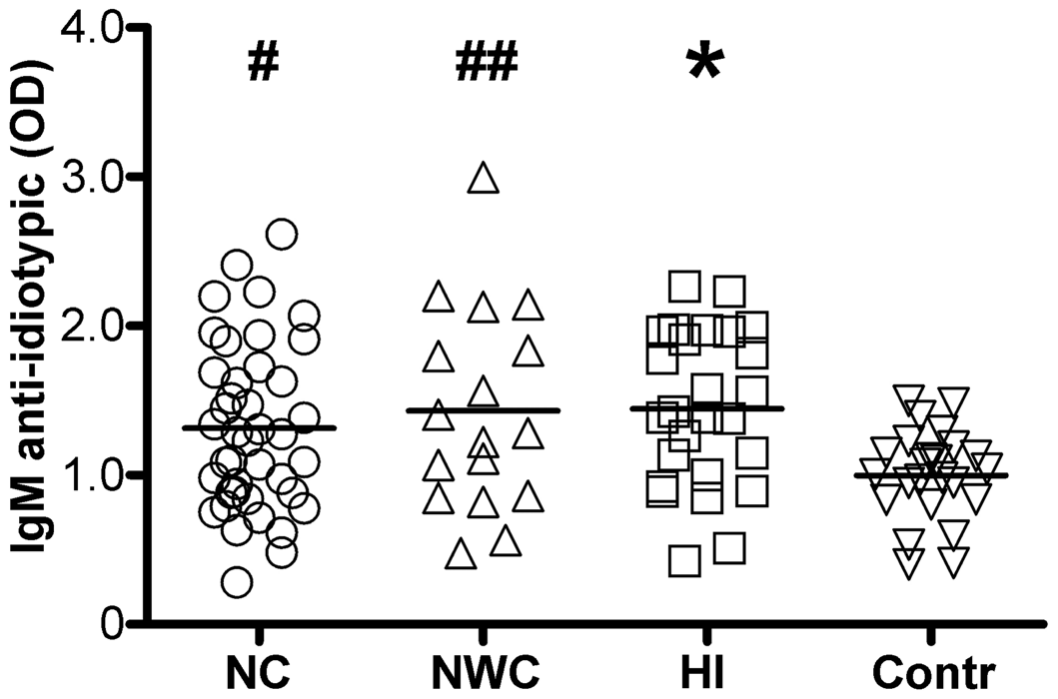
Serum levels of anti-idiotypic IgM autoAbs reactive with hypocretin-1 IgG autoAbs in subjects with central hypersomnia and controls. Bars show means, *p<0.05, Tukey's test vs. controls. # p<0.05 and ## p<0.01, Student's t-test, two-tails vs. controls.

Serum levels of anti-idiotypic IgM autoAbs reacting with hypocretin-1 IgG autoAbs affinity purified from sera of NC patients differed significantly among four groups (ANOVA, p = 0.014). All patient groups showed elevated levels of these anti-idiotypic IgM autoAbs as compared to controls (Fig. 5).

### Correlations of anti-idiotypic IgM autoantibodies with clinical parameters

After correction for multiple tests, we found significant negative correlations between disease duration and levels of anti-idiotypic IgM autoAbs reacting with hypocretin-1 IgG autoAbs (*r = *−0.39, p = 0.004) in all patients' groups combined. Before Bonferroni correction, levels of anti-idiotypic IgM autoAbs correlated negatively with CSF hypocretin-1 levels in all patients' groups (*r = *−0.32, p = 0.03, one-tail), in the NC group (*r = *−0.4, p = 0.04, one-tail) and in the HI group (*r = *−0.7, p = 0.02, two-tails).

## Discussion

The present data revealed altered characteristics of hypocretin-1 reactive autoAbs in subjects with central hypersomnia. We found that levels of hypocretin-1 free IgG autoAbs were lower in NC, NWC and HI groups of patients than in controls. These data are in agreement with a previous report showing low levels of free hypocretin autoAbs in NC patients [15]. Because a fraction of autoAbs exist in immune complexes which are not detectable in normal assays, we measured the levels of total autoAbs reactive with hypocretin-1 peptide using a NaCl-glycine buffer which dissociates immune complexes. With this approach, we found that in contrast to the free fraction of hypocretin-1 autoAbs, total IgG autoAbs are increased in the patients with NC. We acknowledge some limitation of this analysis since few values of total IgG autoAbs had OD above 2.5 which might not be very accurate and could underestimate the actual autoAbs levels. Furthermore, by analyzing the ratios between total and free autoAbs levels which shows proportion of complexed autoAbs, we found that all three groups of patients including NC, NWC and HI had higher percentage of hypocretin-1 autoAbs present in immune complexes than controls. In addition, using hypocretin-1 reactive IgG autoAbs affinity purified from sera samples of NC patients we detected a band that correspond to the hypocretin precursor protein in rat hypothalamic homogenate in Western Blotting. Increased levels of immune complexes of hypocretin-1 IgG autoAbs especially reported in NC may signify deficient hypocretin signalling as has been shown recently for α-melanocyte-stimulating hormone (α-MSH) autoAbs [26]. However, the relevance of systemic hypocretin to the central hypocretin signaling is currently unknown. Peripheral sources of hypocretin, even subject to controversy, may exist [8], [27], [28]. Whether hypocretin-reactive autoAbs are functionally significant remain to be determined as most of immunoglobulins do not cross the blood-brain barrier under physiological condition. Recent study reported altered levels of main subclasses of total IgG in NC and HI patients which further support the involvement of the humoral immune response in central hypersomnia [29].

The composition of immune complexes of hypocretin-1 autoAbs is most likely with anti-idiotypic autoAbs since increased levels of such autoAbs was found in all three groups of patients with central hypersomnia. Although we checked for the IgM class of anti-idiotypic autoAbs, it is probable that other immunoglobulin classes including IgG may form such complexes. The presence of anti-idiotypic autoAbs *per se* is a natural phenomenon constituting the anti-idiotypic network which role is believed to prevent autoimmunity via neutralization of persistent antibodies to endogenous or exogenous antigens [30]. Thus, our finding of elevated levels of anti-idiotypic autoAbs in patients with central hypersomnia may be biologically significant in controlling the peripheral hypocretin availability. Nevertheless, considering the eventual complementarities between the paratope of anti-idiotypic autoAbs and the antigen, which is in this case hypocretin-1 peptide or a cross-reacting protein, one cannot exclude binding of these autoAbs to the hypocretin receptors. Such a phenomenon was shown, for instance, for the anti-idiotypic autoAbs against anti-beta endorphin autoAbs, being able to antagonize beta-endorphin binding to opiate receptors in some subjects with major depression [31]. Whether hypocretin-1 anti-idiotypic autoAbs may interfere with hypocretin signalling relevant to hypersomnia or if they are simply markers of disease progression should be clarified in further studies.

Presence of elevated levels of IgM class of autoAbs normally reflects recent antigenic stimulation [32]. Unexpectedly, we found increased levels of hypocretin-1 total IgM autoAbs and anti-idiotypic IgM autoAbs in all three groups of patients and particularly significant in the HI group. These results may signify a continuous antigenic stimulation during the disease progression, finding supported by a negative correlation noted between disease duration and levels of IgM autoAbs.

Presence of elevated levels of IgA class of autoAbs may reflect luminal, most commonly intestinal or respiratory tracts origin of antigens [33]. We found that NC and HI patients had increased percentage of immune complexes of hypocretin-1 IgA class of autoAbs. This finding may signify that class switch from IgM to IgA hypocretin-1 autoAbs is triggered by a luminal antigen. We previously showed existence of sequence homology between hypocretin-1 peptide and some microorganisms including gut microflora [34] which may account for the concept of molecular mimicry responsible for production of cross-reactive autoAbs [35]. Interestingly, patients with recent onset of NC display elevated anti-streptococcal and Tribbles homolog 2 antibodies [13], [36]. Disease duration correlated positively with levels of IgA hypocretin autoAbs, suggesting an increase of luminal antigenic stimulation to produce such autoAbs during disease progression that again may be triggered by yet unidentified molecular mimicry of microorganisms.

Although several sleep-related characteristics such as daytime sleepiness, sleep onset latency and hypnagogic hallucinations were significantly associated with serum levels of hypocretin-1 autoAbs in patients with central hypersomnia before correction for multiple tests, these associations were not significant after such a correction and, therefore, functional significance of these correlations should be individually verified in further studies. Excessive daytime sleepiness is a hallmark symptom of primary central hypersomnias; however, the neural basis of sleepiness remains unclear in most disorders. Based on the present data, the relevance of hypocretin-1 reactive autoAbs in mechanisms underlying sleepiness may be hypothesized even if the relationship between free/immune-complexed systemic immunoglobulins and the hypothalamic sleep control remain to be clarified.

We may acknowledge several limitations of our study: first, there was no measurement of CSF hypocretin-1 autoAbs. Second, we did not assay serum levels of hypocretin to see their possible correlations with hypocretin autoAbs. Third, patients with HI, NWC and controls were not HLA matched to the patients with NC. The sera was taken after long duration of diseases that has decreased the chance to picking up major alterations of antibody levels with relative overlap even with between-group significant differences. Finally, the number of control subjects was relatively small.

In conclusion, we found that subjects with NC display increased serum levels of hypocretin-1 reactive total IgG autoAbs and all three groups (NC, NWC and HI) show increased serum levels of complex-forming hypocretin-1 autoAbs most likely with anti-idiotypic autoantibodies. Future studies should precise whether hypocretin-reactive autoAbs are involved in physiological regulation of hypocretin availability relevant to arousal and sleep/wakefulness
